# Phospho-proteomic analysis of primary human colon epithelial cells during the early *Trypanosoma cruzi* infection phase

**DOI:** 10.1371/journal.pntd.0006792

**Published:** 2018-09-17

**Authors:** Shankar Suman, Girish Rachakonda, Sammed N. Mandape, Shruti S. Sakhare, Fernando Villalta, Siddharth Pratap, Maria F. Lima, Pius N. Nde

**Affiliations:** 1 Department of Microbiology and Immunology, Meharry Medical College, Nashville, Tennessee, United States of America; 2 School of Graduate Studies and Research, Meharry Medical College, Nashville, Tennessee, United States of America; Universidade Federal de Minas Gerais, BRAZIL

## Abstract

The protozoan parasite *Trypanosoma cruzi*, the causative agent of Chagas disease, causes severe morbidity and mortality in afflicted individuals. About 30% of *T*. *cruzi*-infected individuals present with cardiac, gastrointestinal tract, and/or neurological disorders. Megacolon, one of the major pathologies of Chagas disease, is accompanied by gastrointestinal motility disorders. The molecular mechanism of *T*. *cruzi*-mediated megacolon in Chagas disease is currently unknown. To decipher the molecular mechanism of *T*. *cruzi*-induced alteration in the colon during the early infection phase, we exposed primary human colonic epithelial cells (HCoEpiC) to invasive *T*. *cruzi* trypomastigotes at multiple time points to determine changes in the phosphoprotein networks in the cells following infection using proteome profiler Human phospho-kinase arrays. We found significant changes in the phosphorylation pattern that can mediate cellular deregulations in colonic epithelial cells after infection. We detected a significant increase in the levels of phosphorylated heat shock protein (p-HSP) 27 and transcription factors that regulate various cellular functions, including c-Jun and CREB. Our study confirmed significant upregulation of phospho (p-) Akt S473, p-JNK, which may directly or indirectly modulate CREB and c-Jun phosphorylation, respectively. We also observed increased levels of phosphorylated CREB and c-Jun in the nucleus. Furthermore, we found that p-c-Jun and p-CREB co-localized in the nucleus at 180 minutes post infection, with a maximum Pearson correlation coefficient of 0.76±0.02. Increased p-c-Jun and p-CREB have been linked to inflammatory and profibrotic responses. *T*. *cruzi* infection of HCoEpiC induces an increased expression of thrombospondin-1 (TSP-1), which is fibrogenic at elevated levels. We also found that *T*. *cruzi* infection modulates the expression of NF-kB and JAK2-STAT1 signaling molecules which can increase pro-inflammatory flux. Bioinformatics analysis of the phosphoprotein networks derived using the phospho-protein data serves as a blueprint for *T*. *cruzi*-mediated cellular transformation of primary human colonic cells during the early phase of *T*. *cruzi* infection.

## Author summary

*Trypanosoma cruzi* is a hemoflagellate that is now considered a global health threat in all industrialized regions of the world. Some chagasic patients present with digestive, neurological, and/or cardiac disorders. The mechanisms of *T*. *cruzi*-induced pathology remain to be elucidated. In this study, we challenged primary human colonic epithelial cells with *T*. *cruzi* and evaluated changes in the phosphorylated kinases and phosphoprotein levels that may induce cellular and molecular alterations leading to cellular transformations during the early phase of infection. The parasite induced significant increases in levels of phosphorylated kinases and phosphoproteins that govern multiple cellular pathways associated with immunological, stress, neuronal, and intercellular interactions as well as fibrogenic responses. The parasite also enhanced the levels of p-AKT, p-HSP27, p-JNK, and downstream transcription factors like p-c-Jun and p-CREB during the early infection phase. Additionally, we observed that the phosphorylated transcription factors are translocated to and colocalized in the nucleus in a time-dependent manner. These transcription factors regulate the expression of genes, including genes encoding extracellular matrix proteins, which play a role in the onset of colon pathology observed in some chagasic patients. Our study provides novel insights into the interactome that occurs during acute phase of *T*. *cruzi* infection of primary human colon cells.

## Introduction

The protozoan parasite *Trypanosoma cruzi* is the causative agent of Chagas disease, a neglected tropical disease which causes severe morbidity and mortality worldwide. Originally endemic in South American countries where it still constitutes a severe socioeconomic burden, Chagas disease has spread around the world and become a global health crisis [1, 2]. Currently, the disease is present in all major economically advanced countries due to modern globalization and migration [3]. As many as 30% of afflicted individuals eventually present with cardiac, gastrointestinal tract and/or neurological disorders [4]. The development of megacolon, as one of the pathologies of *T*. *cruzi* infection, is usually accompanied by unwanted changes in gastrointestinal (GI) tract motility which is thought to be due to decrease in the efficiency of the enteric nervous system [5, 6]. GI motility disorders have been attributed to alterations in the number of interstitial cells of Cajal and enteric nervous system defects. Although it is generally agreed that the enteric neurons [7, 8] and interstitial cells of Cajal [8, 9] decrease in numbers in megacolon, it is unclear what roles they play in the pathophysiology of chagasic megacolon. The presence of more natural killer and cytotoxic T-cells in colon lesions from patients with megacolon suggest that immune responses also play a role in the neuronal loss in chagasic megacolon patients [6]. A study using a murine model of chagasic megacolon showed that megacolon was accompanied by increases in colon wall thickness, hypertrophy, and collagen deposition, which are hallmarks of fibrosis [7]. This report correlates with others showing an increase in fibrotic lesions in smooth muscle and myenteric plexus of chagasic megacolon tissue sections [8]. The fibrotic lesions observed in megacolon tissue sections can be caused by increased deposition of extracellular matrix (ECM) and matricellular proteins including TSP-1. The interactions between *T*. *cruzi* and colon cells including colon epithelium cells can deregulate cell signaling pathways leading to increased expression of transcription factors that upregulate the synthesis of ECM proteins [10, 11] causing fibrogenesis and cellular transformation reported in megacolon tissue sections. The role played by colon epithelium in the onset of chagasic megacolon remains unknown.

To understand the pathogenesis of chagasic megacolon, researchers will need to study the role of parasite-induced signaling molecules including cytokines, chemokines, neurotransmitters, and neurotrophic factors in mediating signal transfer within and among colon cells. Currently, studies elucidating parasite-induced changes in colon cells involved in the onset and development of chagasic megacolon is lacking. The fact that there have been reports of Chagas disease outbreaks caused by consumption of *T*. *cruzi* contaminated foods where the infected individuals presented with clinical manifestations comparable to those infected via other types of transmission routes shows that the parasite can infect intestinal epithelial cells [12, 13]. Furthermore, murine experimental *T*. *cruzi* infections have been established using oral cavity and gastrointestinal gavage inoculation routes [14]. We hypothesize that the molecular interactions between *T*. *cruzi* and colon epithelium will deregulate cell signaling cascades, leading to an increase in active transcription factors that can cause increased deposition of matrix proteins, fibrogenesis, loss of colon elasticity and eventually megacolon pathology.

To delineate the molecular mechanisms of *T*. *cruzi*-induced changes in colon epithelium during the early stage of infection, we challenged primary human colonic epithelial cells with invasive *T*. *cruzi* trypomastigotes. This infection model closely mimics the physiological state and changes in host phospho-proteome profile induced by the parasite are essential in understanding parasite mediated pathology.

A predominance of megacolon pathology was reported in *T*. *cruzi* infected individuals from Chile, which is the origin of the Tulahuen strain, suggesting a colon tropism for the strain [15]. Additionally, *T*. *cruzi* Tulahuen strain has been shown to generate experimental murine Chagas disease infection through the gastrointestinal tract [14]. In our study, we show that *T*. *cruzi* Tulahuen trypomastigotes induce significant changes in phosphorylation patterns of a variety of signaling proteins and transcription factors including c-Jun and CREB during the early phase of cellular infection. We also observed that these phosphorylated transcription factors are translocated and colocalized in the nucleus in a time-dependent manner. These *T*. *cruzi* induced changes during the early infection phase can lead to a fibrogenic response in host colon epithelial cells, subsequently resulting in the development of colon pathology.

## Methods

### Antibodies and chemicals

The proteome profiler human phospho-kinase array kit (#ARY003B), as well as antibodies against p-AktT308 (#MAB7419), p-AktS473 (#AF887), c-Jun (#MAB2670), STAT1 (#MAB1490), (RelA/NF kappa B p65 (#MAB5078), p-HSP27 (#MAB23141), HSP27 (#AF1580), TSP-1 (#MAB3074) and SP600125 (Inhibitor) were purchased from R&D Systems (Minneapolis, MN, USA). Antibodies against RSK (#9355S), p-CREBS133 (#9198S), CREB (#9197S), p-JNKT183/Y185 (#9255S), JNK2 (#9258S), p-c-JunS73 (#3270S), and pan Akt (#4691S), as well as p-CREBS133, p-NF-κB p65 (#3031), p-IKKα/β (# 2697), p-STAT1 (#7649), pTAK (#9339), p-IRAK (#11927), IKKα (#2682), p-CREBS133 (87G3) Rabbit mAb Alexa Fluor 647 Conjugate (#14001S), p-JunS73 rabbit mAb with Alexa Fluor 488 conjugate (#12714S), anti-Rb HRP (#7074S), and anti-mouse HRP (#7076S) were purchased from Cell Signaling Technology (Danvers, MA, USA), and beta actin antibody (#sc-69879) from Santa Cruz (Dallas, Texas, USA). SDS (10%), acrylamide (29:1), blotting-grade blocker, precision plus protein marker, 2-mercaptoethanol, 10x Tris/Glycine/SDS, 10x Tris Buffered Saline, 10% Tween 20, Trans-Blot Turbo, Mini Nitrocellulose Transfer Packs were purchased from Bio-Rad Laboratory (Hercules, CA, USA). NP40 cell lysis buffer, Phalloidin and Prolong Gold antifade mounting reagent containing DAPI (#P36935) were purchased from Life Technologies (Carlsbad, CA, USA). The comprehensive kit of human colonic epithelial cells, (HCoEpiC) (#2950) containing primary cells and all required culturing reagents and supplements was purchased from ScienCell Research Laboratory (Carlsbad, CA, USA). Protease inhibitor cocktail set III was purchased from Calbiochem (Gibbstown, NJ, USA). Phosphatase inhibitor cocktails 2 and 3, Trizma, ethyl alcohol, and acetic acid were purchased from Sigma Aldrich (St. Loius, MO, USA).

### Cell lines and cell culture

HCoEpiC were grown in colonic epithelial cell medium (CoEpiCM, Cat. #2951) supplemented with the accompanying supplements as recommended by the manufacturer (ScienCell Research Laboratory, CA). Briefly, tissue culture flasks were incubated with poly L-lysine solution (2 μg/cm^2^) at 37ºC overnight. HCoEpiC were seeded in the coated flasks and cultured at 37ºC and in 5% CO_2_. Confluent cell monolayers (about 80%) were used in our assays. For inhibition of JNK-c-Jun pathway, the cells were pretreated overnight with 100µM SP600125.

### *T*. *cruzi* trypomastigote culture and infection assays

Heart myoblast monolayers (80% confluence) grown in complete DMEM containing glutamax, 10% fetal bovine serum, 1% penicillin/streptomycin, multivitamins, and MEM non-essential amino acids (Life Technologies, Carlsbad, CA, USA) were infected with invasive *T*. *cruzi* trypomastigotes Tulahuen strain clone MMC 20A [16]. Transgenic *T*. *cruzi* Tulahuen trypomastigotes expressing green fluorescent protein (GFP), generated as previously described [17] were used to analyze cellular infection through confocal microscopy. Highly invasive *T*. *cruzi* trypomastigotes were harvested from tissue culture supernatants as previously described [16]. The parasites were washed in Hanks Balanced Salt Solution (HBSS) and resuspended in CoEpiCM without supplements at a concentration of 1x10^7^ parasites/ml. HCoEpiC were starved in CoEpiCM without supplements for 1h at 37°C, 5% CO_2_ prior to the addition of trypomastigotes. The starved cells were challenged with pure population of *T*. *cruzi* trypomastigotes at a ratio of 10 parasites per cell for different time points (60, 90, 120, 180 minutes) essentially as described [10]. Parasites were washed off with 1X DPBS (without calcium/magnesium) and the cells were either processed immediately or stored at -80ºC for further experimentation. To analyze the *T*. *cruzi* infection patterns of HCoEpiC, the GFP trypomastigotes were washed off and the cells were incubated with fresh complete CoEpiCM with daily changes up to 72h. The cells were fixed with 4% paraformaldehyde for 5 minutes at room temperature, washed with 1X DPBS, stained with phalloidin (1:2000) at 4°C overnight and mounted with mounting media containing DAPI to stain the nuclei for microscopy. The number of internalized parasites per cell were evaluated by screening no less than 200 cells per well.

### Human phospho-kinase antibody array

To analyze the phosphorylation profiles of kinases and their protein substrates in *T*. *cruzi*-infected primary colon cells, we used a commercially available human phospho-kinase array kit following the manufacturer's protocol (R&D Systems). This is a membrane-based sandwich immunoassay kit where the capture antibodies against 43 kinase phosphorylation sites, two other proteins and control proteins are spotted in duplicates on nitrocellulose membranes to bind specific target proteins present in the cell lysates (Product Datasheet ARY003B, R&D Systems). Briefly, blocked array membranes were incubated with *T*. *cruzi* infected and the same time points uninfected cell protein lysates, and with the same amount of *T*. *cruzi* trypomastigotes protein lysate (~250µg), at 4°C overnight on a platform shaker. Washed membranes were further incubated with biotinylated antibody cocktails for 2h at room temperature on a rocking platform. The membranes were washed, probed with streptavidin-HRP and visualized by chemiluminescence using X-ray films. The films were scanned, and the density of each spot was quantified against the average of the internal controls as recommended by the manufacturers (R&D Systems). Densitometric data analyses were done using the free Image J software available on the National Institutes of Health (NIH) website (https://imagej.nih.gov/ij/).

### Immunoblotting assays

In order to validate the data obtained using the phospho-kinase array kit, we used immunoblotting assays to evaluate the phosphorylation profile of selected phosphoproteins as previously described [10]. Briefly, serum-starved HCoEpiC incubated with invasive *T*. *cruzi* trypomastigotes were lysed with the NP40 cell lysis buffer containing phosphatase inhibitor cocktails 2 and 3, and protease inhibitor cocktail set III, each at a ratio of 1:100. The cell lysates (20μg/lane) were separated by SDS-PAGE and blotted onto nitrocellulose membranes using the Trans-Blot Turbo system. The membranes were stained with Ponceau S staining solution (#P7170, Sigma Aldrich) to verify protein transfer. The membranes were washed and blocked with blocking buffer (1X TBS pH 7.4, 5% nonfat dry milk and 0.1% Tween-20) for 1h at room temperature. Blocked membranes were incubated with the respective phospho-primary antibodies in antibody buffer (1X TBS pH 7.4, 1% nonfat dry milk and 0.1% Tween-20) at 4°C overnight. The blots were washed to remove excess antibodies, probed with the corresponding HRP-conjugated secondary antibodies, visualized by chemiluminescence, and scanned. The membranes were stripped and re-probed with antibodies against the corresponding total proteins or beta actin to normalize the data. Data were collected from three independent sets of experiment and analyzed by densitometry using Image J software.

### Immunofluorescence assays

Immunofluorescence assays were used to detect phosphoproteins of interest in the nucleus. HCoEpiC seeded on Lab-Tek chamber slides were used for immunofluorescence assays as previously described [10]. Briefly, *T*. *cruzi* trypomastigotes (10 parasites per cell) were incubated with the cells for various lengths of time. The parasites were washed off. The cells were fixed with 4% paraformaldehyde for 5 minutes at room temperature and washed with 1X DPBS. Fixed cells were perforated with 0.1% Triton-X100 in TBS for 5 minutes and blocked with 3% BSA-PBS for 30 minutes at room temperature. Slides were incubated with anti-human-p-c-JunS73 Alexa 488 conjugate (1:100), anti-p-CREB Alexa 647 conjugate (1:100), and phalloidin (1:2000) at 4°C overnight. The slides were washed with 1% BSA-PBS and mounted with mounting media containing DAPI to stain the nuclei. For colocalization assays, the fixed, perforated and blocked slides were incubated with a mixture containing anti-human-p-c-JunS73 Alexa 488 conjugate and anti-human-p-CREB Alexa 647 conjugate at a dilution of 1:100 each, washed and mounted with mounting media containing DAPI to stain the nuclei. Stained slides were analyzed using the Nikon A1R confocal microscope at the Morphology Core Facility at Meharry Medical College.

### Mapping of biological pathway interactions

The functional analysis of biological pathways of altered phosphoproteins were elucidated using bioinformatics approach. Protein annotation was done using GENECARDS (www.genecards.org) and DAVID (david.ncifcrf.gov) to find common reference and to achieve de-aliasing. Biological Pathways were constructed using Ingenuity Pathway Analysis Path Explorer (IPA, QIAGEN). Significantly enriched canonical pathways were determined using Fisher’s Exact test for enrichment. Significantly enriched canonical pathways constructed by IPA were grouped into five meta-level categories: 1) immunological response, 2) fibrotic extracellular signaling, 3) neuronal response, 4) intracellular signaling, and 5) stress associated response. Visualization of proteins to pathway group mapping was done using table2net (http://tools.medialab.sciences-po.fr/table2net/) for graph file parsing and formatting followed by Gephi (https://gephi.org/) for visualization.

### Statistical analysis

All data were collected from three independent sets of experiments. Alterations in phosphorylated protein levels were analyzed using Student’s *t*-test or one-way analysis of variance (ANOVA) for multiple groups of data. The statistical analyses were performed using SPSS software for phospho-array measurements. For altered protein expression, a fold change of ≥1.5 with p-value ≤ 0.01 was considered significant. For biological pathway analysis and mapping, a Fisher’s exact test was used to identify enriched pathways and a p-value ≤ 0.001 was considered significant.

### List of gene and Entrez ID of all proteins used in the manuscript

AKt1/2/3(AKT1 [207], AKT2 [208], AKT3[10000]), AMPKα1 (PRKAA1 [5562]), AMPKα2 (PRKAA2 [5563]), Chk2 (CHEK2 [11200), c-Jun (JUN [3725), CREB (CREB1 [1385]), EGFR (EGFR [1956]), Enos (NOS3 [4846]), ERK1/2 (MAPK1 [5594]), FAK (PTK2 [5747]), Fgr (FGR [2268]), Fyn (FYN [2534]), GSK-3α/β (GSK3A [2931], GSK3B [2932]), HcK (HCK [3055]), HSP27 (HSPB1 [3315], HSPB2 [3316]), HSP60 (HSP60 [3329]), JNK1/2/3 (MAPK8 [5599], MAPK9 [5601], MAPK10[5602]), Lck (LCK [3932]), Lyn (LYN [4067]), MSK1/2 (RPS6KA5 [9252], RPS6KA4 [8986]), p27 (IFI27 [3429]), p38α (MAPK14 [1432]), p53 (TP53 [7157]), p70 S6 (RPS6KB1 [6198], RPS6KB2 [6199]), PDGF Rβ (PDGFRB [5159]), PLC-γ1 (PLCG1 [5335]), PRAS40 (AKT1S1 [84335]), PYK2 (PTK2B [2185]), RSK1/2/3 (RPS6KA1 [6195], RPS6KA3 [6197], RPS6KA2 [6196]), Src (SRC [6714]), STAT2 (STAT2 [6773]), STAT3 (STAT3 [6774]), STAT5a (STAT5A [6776]), STAT5a/b (STAT5A [6776], STAT5B[6777]), STAT5b (STAT5B [6777]), STAT6 (STAT6 [6778]), TOR (MTOR [2475]), WNK1 (WNK1 [65125]), Yes (YES [7525]), β-Catenin (CTNNB1 [1499]), TSP1 (THBS1 [7057]), β-ACTIN (ACTB [60]), IKKβ (IKBKB [3551]), IRAK4 (IRAK4 [51135]), TAK-1 (MAP3K7 [6885]), JAK2 (JAK2 [3717]), STAT1 (STAT1 [6772]), NFKβ-P65 (RELA [5970]), IKKα (CHUK [1147]).

## Results

### Kinetics of HCoEpiC infection by *T*. *cruzi* Tulahuen strain

**Fig 1:**
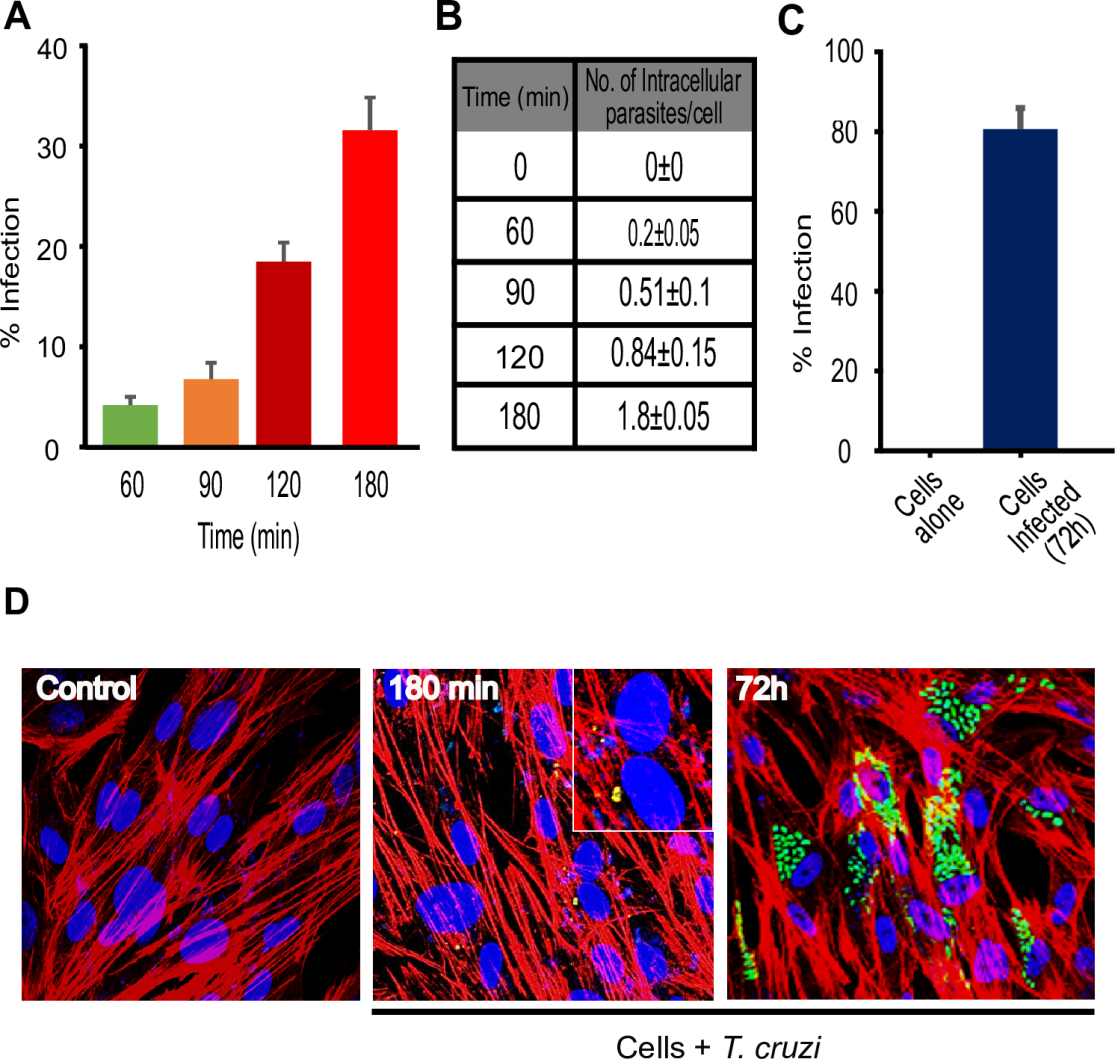
Characterization of human colonic epithelial cells *in vitro* infection by *T*. *cruzi* Tulahuen trypomastigotes (MMC 20A). Primary HCoEpiC cells were infected with transgenic *T*. *cruzi* trypomastigotes, Tulahuen strain (MMC20A) expressing GFP to reveal the infectivity profile. HCoEpiC were grown in Lab-Tek chamber slides (~80% confluence), starved in CoEpiCM without supplements and challenged with transgenic *T*. *cruzi* trypomastigotes during 0, 60, 90, 120, and 180 minutes. Parasites were washed off and the slides were fixed to evaluate internalized parasites. For regular infection, incomplete CoEpiCM was replaced with complete medium containing supplements and slides incubated in tissue culture incubator. The complete media was changed daily until 72 hours post infection. The slides were washed, fixed, stained with phalloidin and DAPI before being subjected to confocal microscopy analysis where average cellular infections were evaluated in several fields. (A) The percentage of infected colonic epithelial cells at 0, 60, 90, 120, and 180 minutes (B) Chart showing the number of intracellular parasites per cell (mean ± SE) 0, 60, 90, 120, and 180 minutes of infection (C) The percentage of infected colonic epithelial cells at 72 h of regular infection (D) Microscopic images of control and *T cruzi* trypomastigotes infected HCoEpiC at 180 min and 72h.

To evaluate *T*. *cruzi* invasiveness of HCoEpiC, we analyzed the percentage of infection after challenging the cells with transgenic *T*. *cruzi* Tulahuen trypomastigotes expressing GFP at different time points. The percentage of infected HCoEpiC was maximum at 180 minutes, where about 30% of the cells are infected with 1.8±0.05 parasites per cell (Fig 1A and 1B). HCoEpiC sustained regular *T*. *cruzi* infection where we observed that more than 80% of the cells contained multiplying amastigotes at 72h post infection (Fig 1C). The data is represented microscopically (Fig 1D). Our data represent the first report of the kinetics of primary human colon epithelial cells *in vitro* infection by *T*. *cruzi* Tulahuen strain.

### The unique phosphoproteomic pathway signature during the early phase of *T*. *cruzi* infection

**Fig 2:**
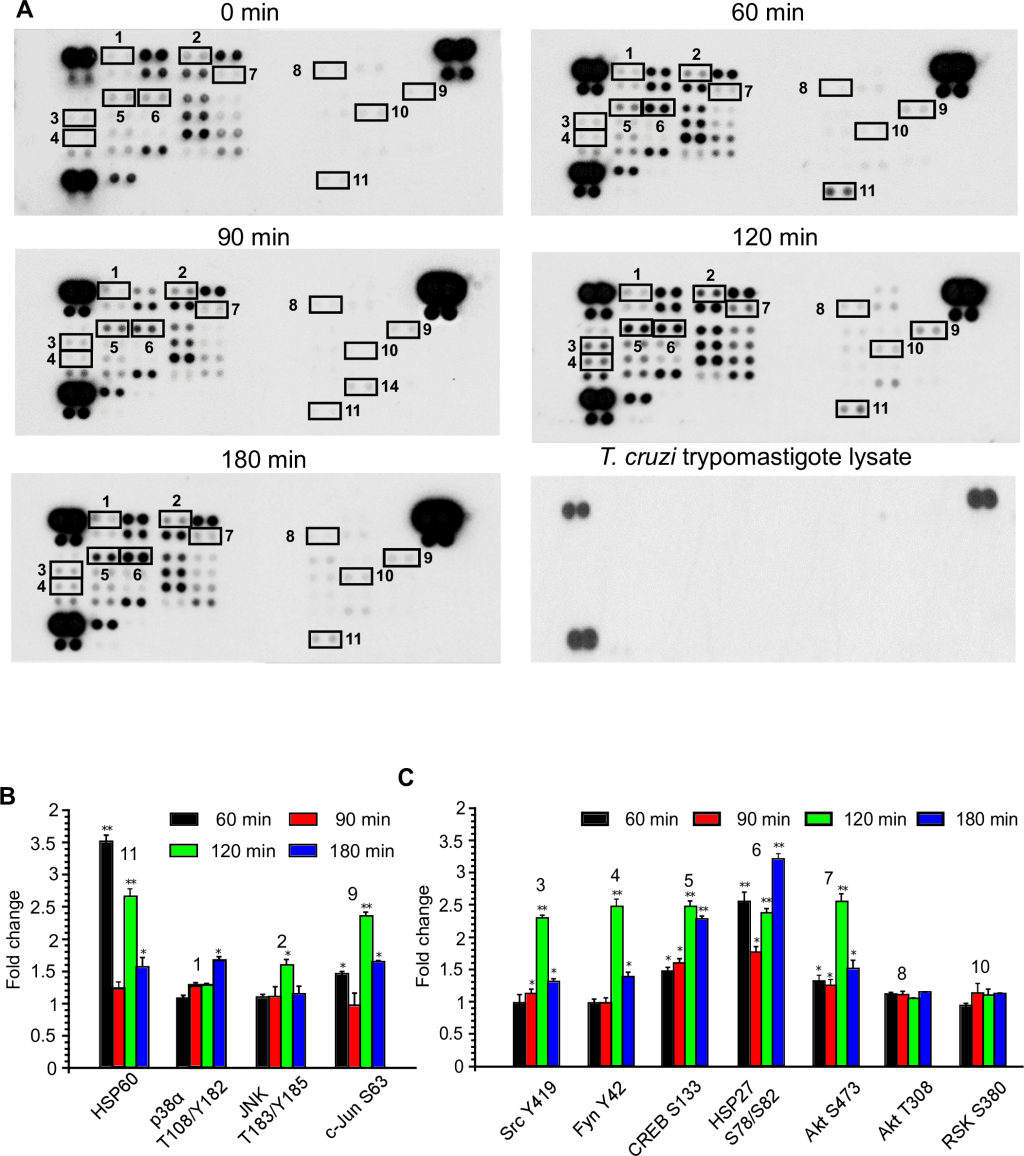
Phosphoproteomic array analysis of *T*. *cruzi*-infected human colonic epithelial cells. Primary HCoEpiC challenged with *T*. *cruzi* at multiple time points were lysed and incubated with phosphoproteomic array membranes. (A) Template showing location of kinases and phosphoprotein antibodies spotted onto the human phospho-kinase array membrane. The signals of selected kinases and phosphoproteins at 0, 60, 90, 120, and 180 minutes are indicated by numbers (1–11). Equal amount of *T cruzi* trypomastigotes lysate was used to evaluate cross reactivity with the membranes. Each signal number was maintained throughout the time course of the experiment. Reference spots are included to align the array membrane and to show that the array has been incubated with streptavidin-HRP. (B) Quantification of mean spot pixel density relative to control represented as fold change for proteins associated with cellular transformation pathways. (C) Quantification of mean spot pixel density relative to control for proteins associated with other cellular pathways. Mean values of biological replicates ± SE are shown. The value of p<0.05 was considered significant. *p<0.05; **p<0.001.

To gain insight into the early regulation of phosphoproteins and their associated signaling cascades mediated by *T*. *cruzi* trypomastigote infection in colonic epithelial cells, we utilized the human phospho-kinase array. We challenged HCoEpiC with *T*. *cruzi* for different lengths of time (0, 60, 90, 120 and 180 minutes) and analyzed the protein phosphorylation profiles following infection (Fig 2A, S1 Table). The time intervals selected were adequate to accommodate important downstream phosphorylation patterns. We also analyzed the protein phosphorylation profiles of HCoEpiC in the starved condition in the absence of parasites at the same time points. The phosphorylation profile of the 43 kinase phosphorylation sites was conserved over time in uninfected cells and did not affect the *T*. *cruzi* induced phosphorylation pattern (S1 Fig, S2 Table). Furthermore, we also observed no cross reactivity on the membranes using the same amount of *T*. *cruzi* trypomastigote lysate (Fig 2A). The reference spots on the arrays (Fig 2A and S1 Fig) are included to align the array membrane and to show that the array has been incubated with streptavidin-HRP during the assay procedure. We found that during the early phase of infection, *T*. *cruzi* infection significantly altered the phosphorylation pattern of 21 kinase phosphorylation sites (S1 Table). Our interest here is to group and map the altered phosphoproteins based on their functional role; including fibrosis, neurological signaling, and immune signaling among others. We employed bioinformatics analyses to determine the deregulated pathways associated with modulated phosphoproteins. Our analyses uncovered several phosphoproteins that are involved with cellular transformation pathways as well as other functional pathways associated with proinflammatory responses and several disease pathogenesis pathways. Specifically, we found that the parasite activated the JNK and c-Jun signaling pathways (Fig 2B) and CREB associated signaling pathway (Fig 2C). The level of p-c-Jun was significantly increased by 2.4±0.06 fold at the 120-minutes but it is 1.7±0.02 at 180 minutes compared to control. Phosphorylation of JNK2, a known upstream molecule of c-Jun, was also upregulated during infection. The phosphorylation of transcription factor CREB was significantly increased by at least two-fold compared to the control. This was accompanied by a significant increase in the phosphorylated levels of HSP27, as well as kinases Src, Akt S473 and Fyn at different time points. The levels of p-Akt T308 and p-RSK were not significantly increased (Fig 2C). Additionally, phosphorylated levels of several proteins including ERK1/2, p27, TOR and STAT5A were downregulated at differing time points (S1 Table). Taken together, these results provide us with the foundation to map the phospho-proteomic network that operates in human colon cells challenged with *T*. *cruzi*.

### Bioinformatics analysis of biological network interactions among the modulated phosphoproteins during *T*. *cruzi* infection

**Fig 3:**
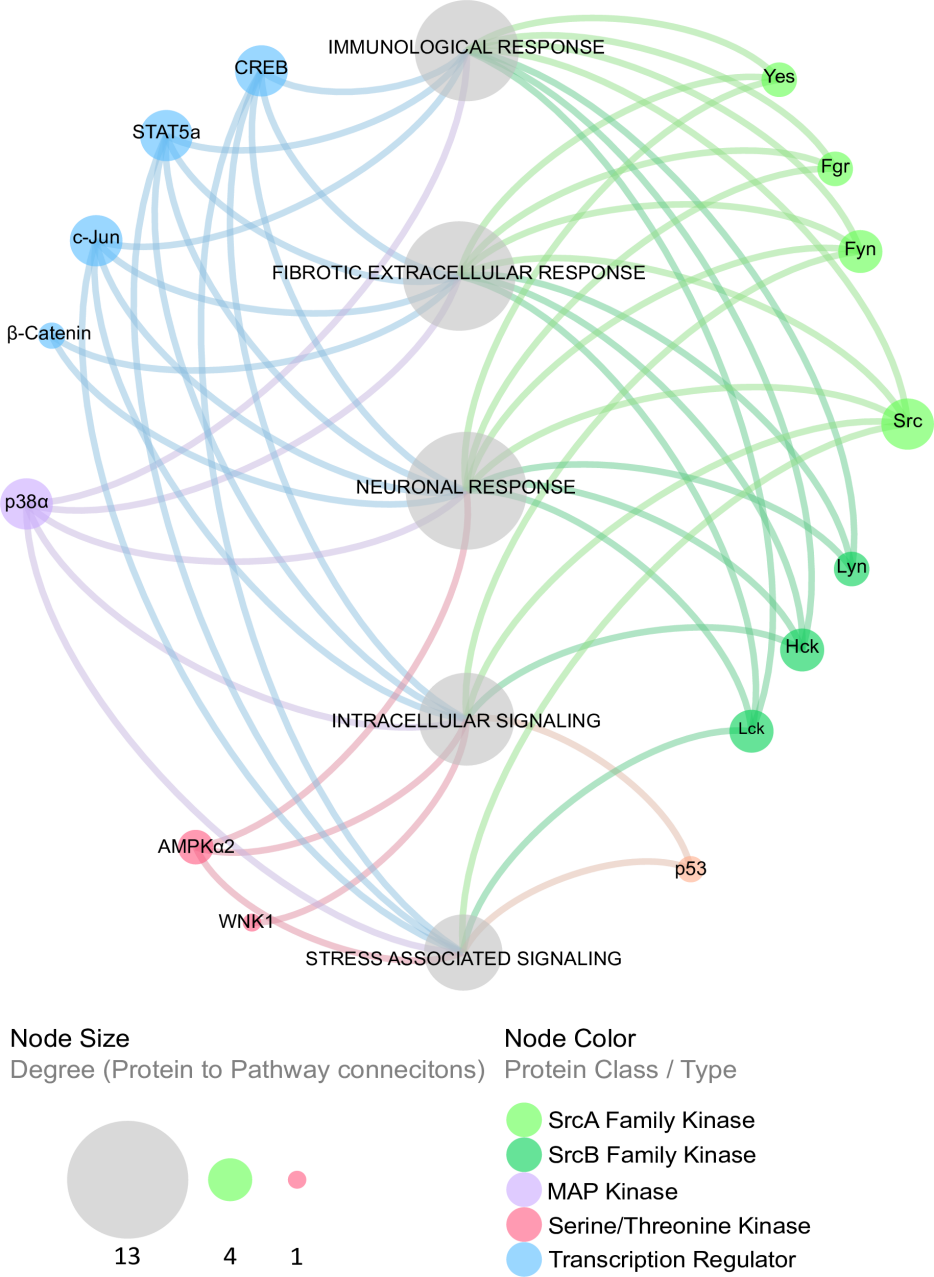
The phosphoprotein biological interaction network in *T*. *cruzi*-infected cells. Cellular pathways associated with the altered phosphoproteins as a result of *T*. *cruzi* infection were derived and grouped at the meta-level using enrichment analysis of significant protein changes mapped to the following canonical pathway groups: immunological response, fibrotic extracellular response, neuronal response, intracellular signaling, and stress-associated signaling. Proteins were categorized into type or class (node color) and degree of connection to their respective cellular signaling pathways (node size).

To understand the functions of the altered phosphoproteins during *T*. *cruzi* infection and their role in Chagas disease progression, we developed a biological interaction network that to indicate the relationship among individual proteins in the colon cells as mediated by *T*. *cruzi*. We were able to map the phosphoproteins to multiple cellular processes and pathways that are deregulated in many disease states (Fig 3). Specifically, we mapped the altered phosphoproteins to stress, immunological, intracellular, fibrotic, and neuronal signaling responses, indicating the interaction among different yet overlapping arms of cellular responses that can contribute to the onset of colon pathology induced by *T*. *cruzi* infection. The canonical pathways involved during the early phase of *T*. *cruzi* infection of primary HCoEpiC is shown (S3 Table).

### Early *T*. *cruzi* infection increases p-CREB in colonic epithelial cells

**Fig 4:**
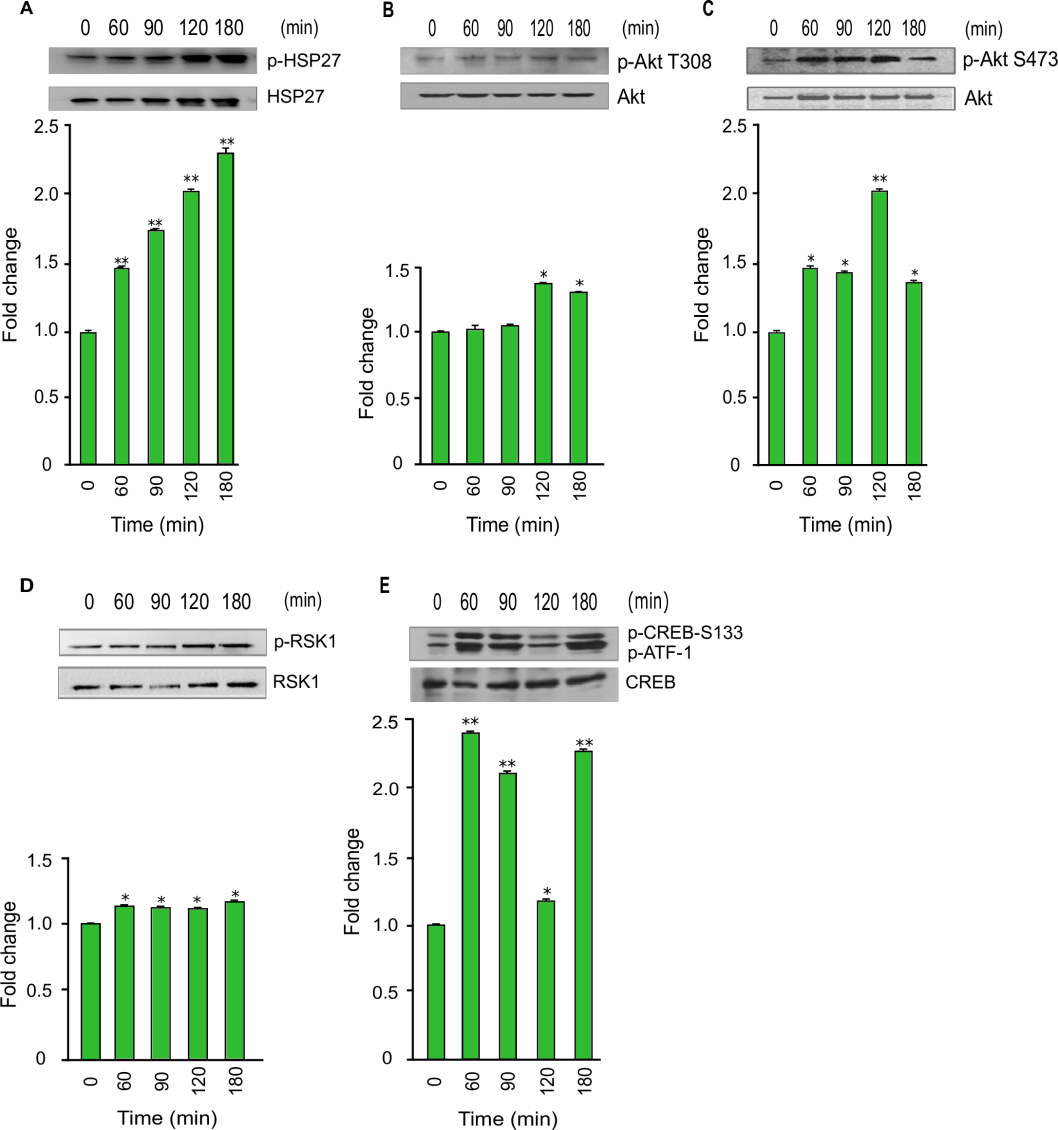
Phosphorylation of Akt, RSK, and CREB during *T*. *cruzi* infection. Lysates from primary HCoEpiC infected with *T*. *cruzi* at multiple time points were resolved by SDS-PAGE, blotted, and probed with antibodies against (A) p-HSP27 (B) p-Akt T308, (C) p-Akt S473, (D) p-RSK, and (E) p-CREB, and developed as described. The blots were stripped and reprobed with antibodies against the respective total proteins. The blots were developed by chemiluminescence and scanned. The normalized fold change in the level of each phosphorylated protein was determined and plotted in the bar graph below the respective blot. The bar graphs represent mean values ± SE from three independent biological replicates. The value of p<0.05 was considered significant; *p<0.05 **p<0.001.

The phosphoproteomic array revealed the upregulation of p-CREB in the host cells. The array data also showed that the phosphorylated levels of Akt S473 and HSP27 were significantly upregulated. We found that the level of pHSP27 was increased more than two-fold at 120 and 180 min (Fig 4A). Our western blot data showed that the phosphorylated level of Akt T308 was increased to 1.34±0.03 fold at 120 minutes and then decreased to 1.27±0.01 fold at 180 minutes (Fig 4B). The phosphorylated level of Akt S473 was significantly upregulated at all-time points during the infection to a maximum of 2.07±0.04 fold at 120 minutes, and then decreased to 1.38±0.11 at 180 minutes (Fig 4C). Our data also showed that the regulatory and kinase domains of Akt were activated by *T*. *cruzi* infection. These results indicate that the Akt signaling pathway in the host cells were activated by the parasite in the early infection phase. Furthermore, our array data also showed that pRSK was upregulated in the infected host cells. Our western blot data revealed a steady gradual upregulation of pRSK to 1.19±0.01 fold at 180 minutes compared to uninfected control cells (Fig 4D). Since p-Akt and p-RSK are upregulated and they are upstream signaling molecules of CREB, we decided to validate the fold change of phosphorylated CREB. Immunoblot analysis of cellular lysates showed that p-CREB was significantly upregulated at least two-fold at several time points (Fig 4E).

### *T*. *cruzi* elevates the activation signals of pro-inflammatory responses

**Fig 5:**
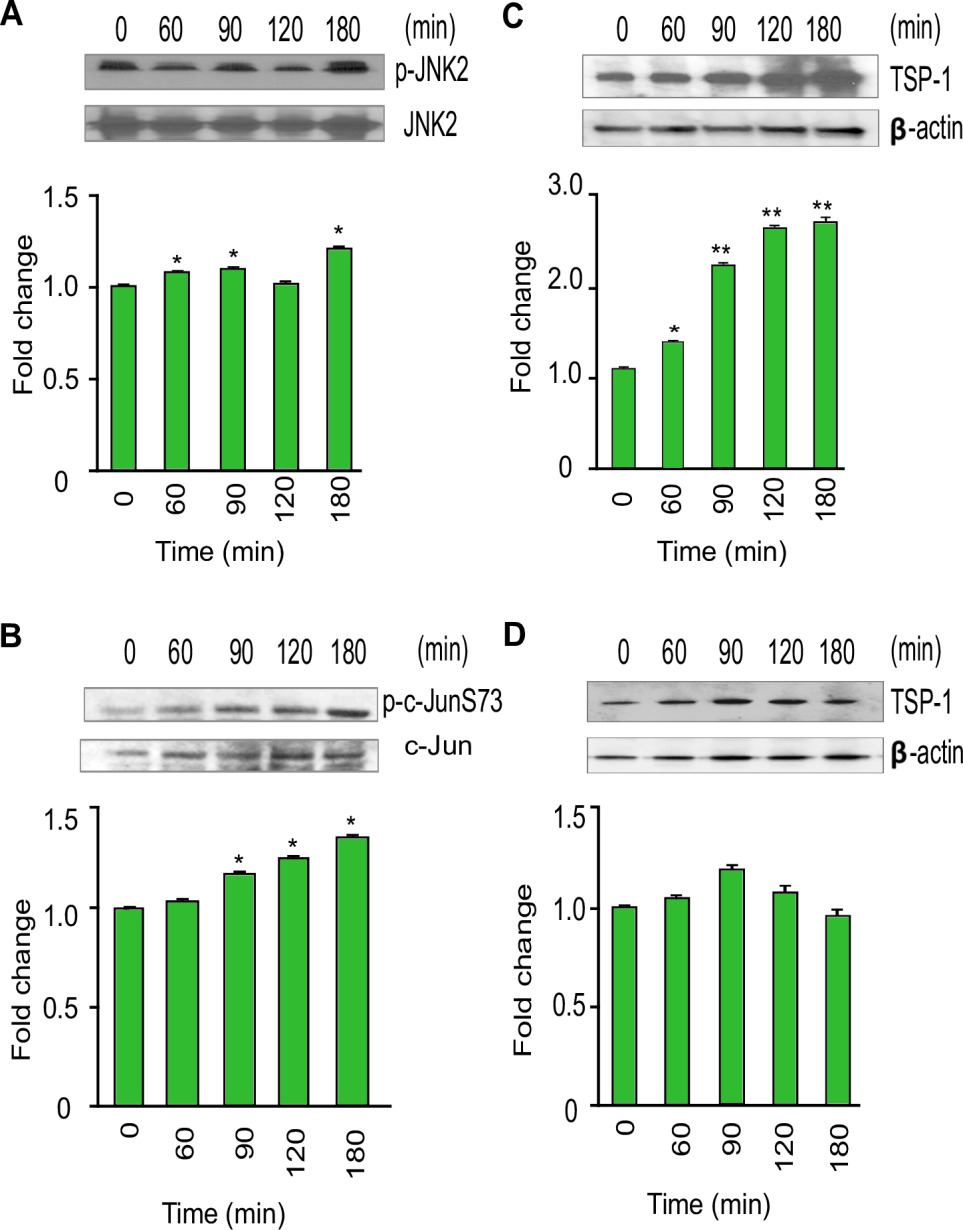
*T*. *cruzi* induces increases in TSP-1 and phosphorylated levels of JNK and c-Jun in HCoEpiC. Lysates from primary HCoEpiC infected with *T*. *cruzi* at multiple time points were resolved by SDS-PAGE, blotted, and probed with antibodies against (A) p-JNK2, (B) p-c-JunS73, and (C) TSP-1 (D) TSP-1 in HCoEpiC treated with SP600125 and developed as described. The blots were stripped, reprobed with antibodies against the respective total proteins or beta actin and developed by chemiluminescence. The developed films were scanned. The normalized fold change in the level of each phosphorylated protein was determined and plotted in the bar graph below the respective blot. The bar graphs represent mean values ± SE from three independent biological replicates. The value of p<0.05 was considered significant. *p<0.05; **p<0.001.

**Fig 6:**
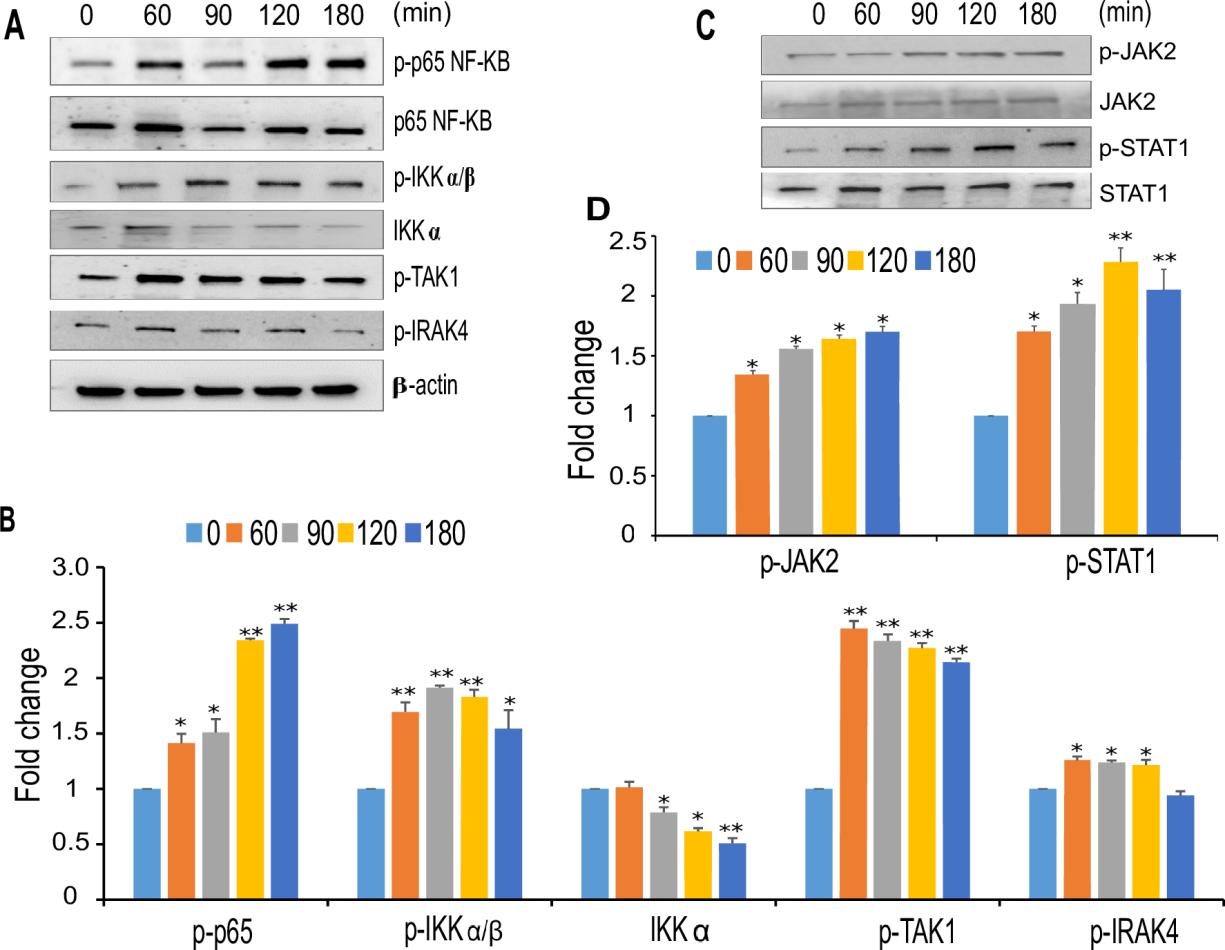
Activation of pro-inflammatory pathways during *T*. *cruzi* infection of HCoEpiC. Lysates from primary HCoEpiC infected with *T*. *cruzi* at multiple time points were resolved by SDS-PAGE, blotted, probed with antibodies against (A) p-p65 NF-KB, p-IKKα/β, pTAK1, pIRAK4 and developed as described. The blots were stripped, reprobed with antibodies against the respective total proteins or beta actin and developed by chemiluminescence and scanned. (B) The normalized fold change of each protein was determined and plotted in the bar graph (C) p-STAT1 and p-JAK2 and developed as described. The blots were stripped and reprobed with antibodies against the respective total proteins. The blots were developed by chemiluminescence and scanned. (D) The normalized fold change in the level of each phosphorylated protein was determined and plotted in the bar graph. The bar graphs represent mean values ± SE from three independent biological replicates. The value of p<0.05 was considered significant; *p<0.05 **p<0.001.

The phosphoproteome data analysis and the bioinformatics analysis of network interactions revealed that *T*. *cruzi* infection promotes the activation of pro-inflammatory signaling pathways. We are particularly interested in JNK signaling, a pathway that is well-known to be activated by external stimuli but has yet to be explored in Chagas disease progression. Phosphorylation of c-Jun, which is downstream of JNK signaling, can mediate cellular transformations implicated in many severe disease states. Our phospho-array data showed significant increases in the level of phosphorylated JNK and c-JunS63 in *T*. *cruzi*-infected colonic cells. We evaluated the levels of phosphorylated JNK and c-JunS73 (not present in the array) at various time points by western blot analysis using lysates of colonic epithelial cells challenged with *T*. *cruzi*. Our immunoblot data showed that the level of phosphorylated JNK, an upstream molecule of c-Jun, was upregulated by 1.24±0.02 fold at 180 minutes compared to uninfected control colonic cells (Fig 5A). The level of p-c-JunS73 was also upregulated at 90, 120 and 180 minutes compared to control cells (Fig 5B). Hence, the western blot results match with the phospho-array data. To evaluate whether *T*. *cruzi* infection in these cells lead to changes in the expression of c-Jun target genes, we analyzed the level of thrombospondin-1 (TSP-1) protein in the infected cells at the different time points. We observed that the level of TSP-1 protein increases with the infection of HCoEpiC up to a maximum of 2.78±0.05 fold at 180 minutes compared to uninfected control (Fig 5C) indicating that the expression of TSP-1 protein is increased during *T*. *cruzi* infection of colon epithelial cells. To confirm this, we preincubated the cells with SP600125 (100µM), a pharmacological inhibitor of the JNK-c-Jun pathway and observed a decrease in p-JNK and p-c-Jun (S2 Fig). The amount of downstream TSP-1 in the cells pretreated with the inhibitor was significantly decreased in the presence of *T*. *cruzi* (Fig 5D). In order to evaluate the kinetics of *T*. *cruzi* induced proinflammatory responses in HCoEpiC, we investigated the regulation of NF-kB associated signaling molecules in *T cruzi* infected HCoEpiC. We found that the p-p65 level was increased up to a maximum of 2.48±0.04 at 180 minutes compared to uninfected control (Fig 6A and 6B). The level of p-IKKα/β was significantly increased to a maximum of 1.91±0.02 at 90 minutes with a corresponding decrease in IKKα levels to a minimum of 0.59±0.05 at 180 minutes (Fig 6A and 6B). Furthermore, we analyzed p-TAK1 and p-IRAK4, upstream regulators of NF- kB pathway. We observed that p-TAK1 was increased to a maximum of 2.30±0.08 at 60 minutes and p-IRAK4 was upregulated by *T*. *cruzi* infection to a maximum of 1.43±0.07 at 60 minutes (Fig 6A and 6B). We also evaluated the levels of p-JAK2 and p-STAT1, signaling molecules that play roles in the regulation of interferon signaling pathway. We observed that levels of p-JAK2 increased to a maximum of 1.73±0.07 at 180 minutes and that of p-STAT1 increased to a maximum of 2.21±0.11 at 120 minutes in *T*. *cruzi* infected HCoEpiC (Fig 6C and 6D).

### Phospho-CREB and p-c-Jun are translocated to the nucleus during the early phase of *T*. *cruzi* infection

**Fig 7:**
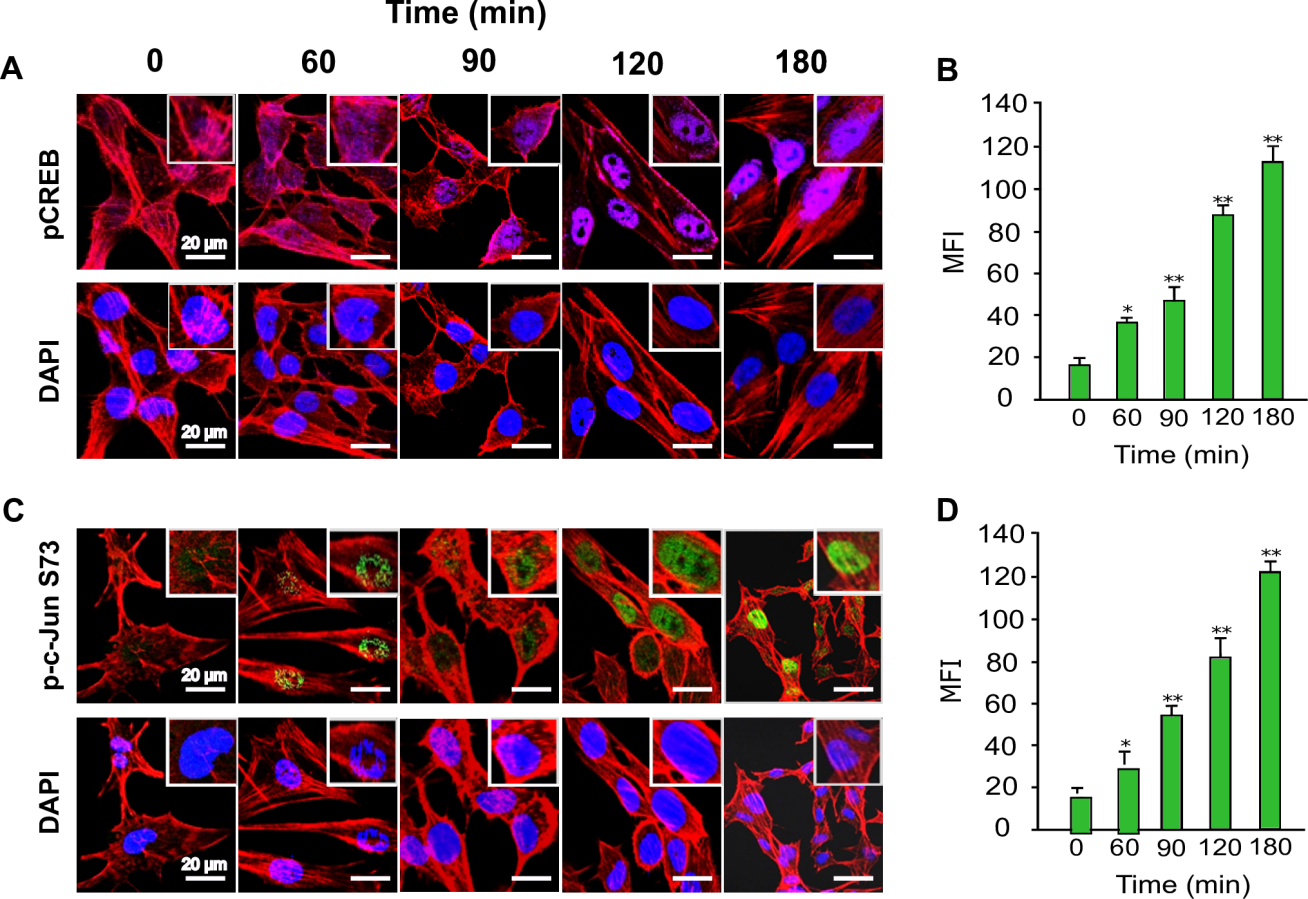
Translocation of transcription factors p-CREB and p-c-Jun into the nuclei of colon epithelial cells during *T*. *cruzi* infection. Primary HCoEpiC grown on Lab-Tek chamber slides were challenged with *T*. *cruzi* at multiple time points, washed, fixed, perforated with 0.1% Triton-X100, blocked with 3% BSA-PBS, and incubated at 4°C overnight in solutions containing phalloidin and one of the following antibodies: (A) anti-human p-CREB-Alexa 647, (C) anti-human-p-c-JunS73-Alexa 488. The slides were washed and mounted with mounting media containing DAPI to stain the nuclei. Stained slides were analyzed by confocal microscopy and the mean fluorescence intensity (MFI) values were plotted for (B) p-CREB, (D) p-c-JunS73. Scale bar, 20 μm. Each confocal microscopy image is a representative of three independent biological replicates. The bar graphs represent MFI values ± SE from three independent biological replicates. The value of p<0.05 was considered significant. *p<0.05; **p<0.001.

Our phospho-array data showed that p-c-Jun and p-CREB were significantly upregulated in colonic cells during the early phase of *T*. *cruzi* infection. These increase in the phosphorylation of several key proteins was validated by immunoblot assays. To understand the importance of upregulated p-CREB and p-c-Jun in colonic epithelial cells during early *T*. *cruzi* infection, we performed immunofluorescence assays to evaluate nuclear translocation and colocalization of both transcription factors. To do this, we measured the localization of each phosphoprotein using the mean fluorescence intensity (MFI). Our data showed that p-CREB was translocated to the nucleus. Specifically, the nuclear translocation of p-CREB significantly increased over time to a maximum of 112.6±10.9 MFI at 180 minutes (Fig 7A and 7B). We also performed immunofluorescence assays to evaluate the nuclear translocation of p-c-JunS73. Our results show nuclear staining of this phosphoprotein during early phase of *T*. *cruzi* infection. We found that the nuclear localization of p-c-JunS73 significantly increased over time to a maximum of 123.2±7.5 MFI at 180 minutes (Fig 7C and 7D). These data agree with our array analysis that indicates that *T*. *cruzi* infection increased the level of active transcription factors p-CREB and p-c-JunS73 in the nuclei of infected primary human colon cells.

**Fig 8:**
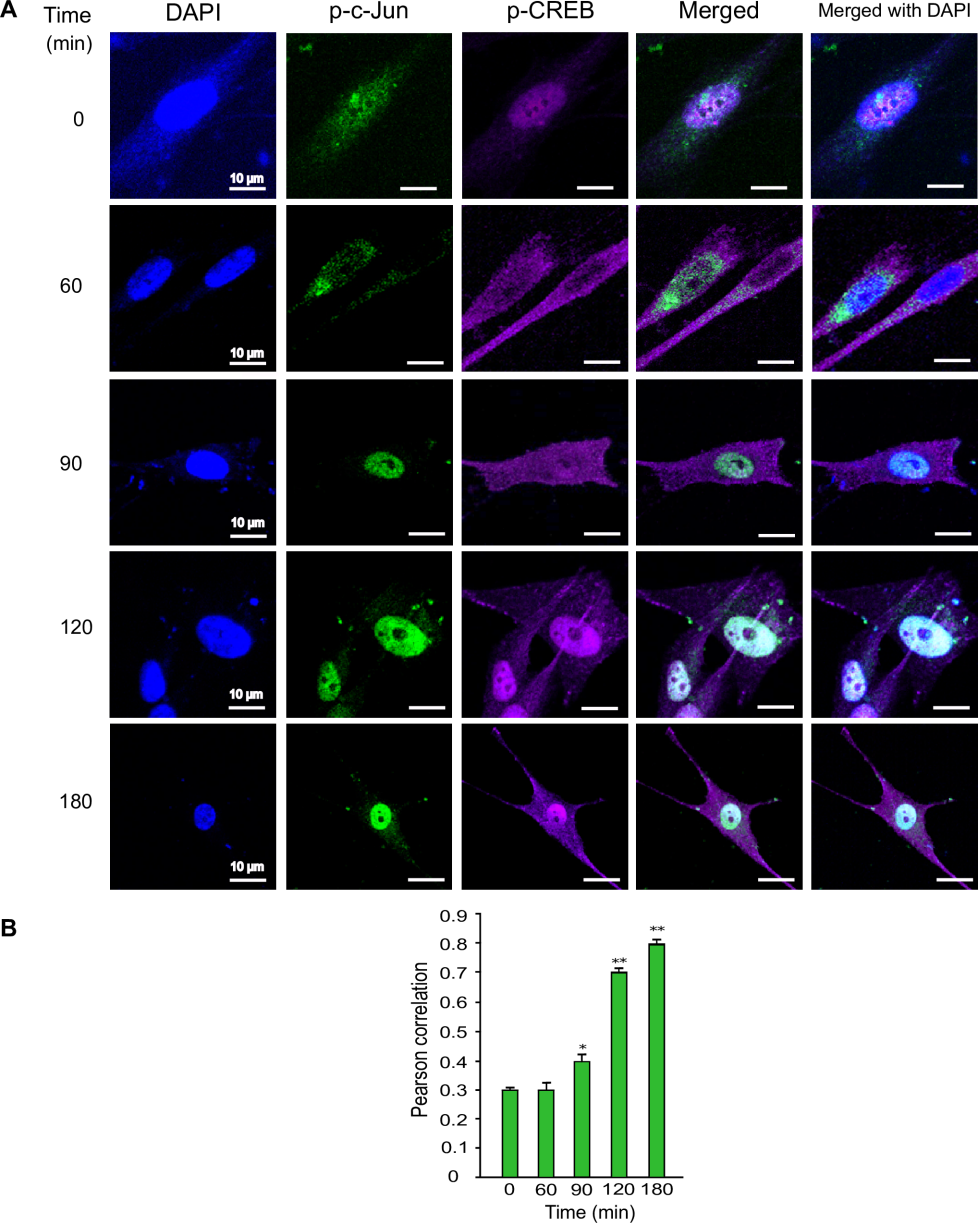
Colocalization of p-CREB and p-c-Jun in the nuclei of primary colon epithelial cell during *T*. *cruzi* infection. Primary HCoEpiC grown on Lab-Tek chamber slides were challenged with *T*. *cruzi* at multiple time points, washed, fixed, perforated with 0.1% Triton-X100, blocked with 3% BSA-PBS, and probed with a mixture of anti-human-p-c-JunS73-Alexa 488 and anti-human-p-CREB-Alexa 647, each at a dilution of 1:100, at 4°C overnight (A). The slides were washed, and mounted with mounting media containing DAPI to stain the nuclei. Stained slides were analyzed by confocal microscopy. Each confocal microscopy image is a representative of three independent biological replicates. Scale bar, 10 μm. (B) The fluorescence intensities of the merged antibody signals were analyzed using confocal microscopy software to generate Pearson correlation coefficients. The bar graphs represent Pearson correlation coefficients values ± SE from three independent biological replicates. The value of p<0.05 was considered significant. *p<0.05; **p<0.001.

Since we observed that the nuclear translocation of p-c-Jun and p-CREB are increasing with time during *T*. *cruzi* infection, we next evaluated if both active transcription factors colocalized in the nucleus. Our confocal microscopy data showed that both phosphorylated transcription factors also colocalized in the nucleus, and that the extent of this colocalization increased over time during *T*. *cruzi* infection (Fig 8A and 8B). The Pearson’s correlation value of this colocalization significantly increased with time to 0.70±0.02 at 120 minutes and a maximum of 0.76±0.02 at 180 minutes (Fig 8B). Taken together, these results show for the first time that *T*. *cruzi* infection enhances the colocalization of both transcription factors into the nuclei of infected colon cells.

## Discussion

Chagasic megacolon is a major pathology associated with severe morbidity and mortality in *T*. *cruzi*-infected patients. The molecular mechanisms that cause megacolon in Chagasic patients remains largely undefined. Primary colonic epithelial cells constitute a good model for studying the mechanisms of *T*. *cruzi* infection of colon cells. We found that *T*. *cruzi* can infect colonic epithelial cells with more than 80% cellular infection after 72h (Fig 1). We hypothesize that during *T*. *cruzi* infection, the parasite deregulates host signal transduction and eventually the gene transcription profiles to cause symptoms associated with Chagas disease [10, 18, 19]. To elucidate the molecular mechanism of *T*. *cruzi*-induced molecular alterations in colon cells, we challenged primary human colonic epithelial cells with invasive *T*. *cruzi* trypomastigotes for various lengths of time (0, 60, 90, 120 and 180 minutes) to evaluate the pattern of altered phosphoproteins after infection using phospho-proteomic arrays and the mapping of biological network interactions. Analyzing the phosphorylation profiles of kinases and their protein substrates is essential for understanding how cells recognize and respond to the presence of *T*. *cruzi* in their micro-environment. Normal cellular activities in colon cells are highly susceptible to alterations following exposure to *T*. *cruzi* trypomastigotes. The analysis of phosphorylation profiles of kinases and their respective protein substrates may facilitate our understanding of the cellular response mediated by *T*. *cruzi* trypomastigotes in host colonic cells. In the present study, our goal was to decipher the phospho-signaling pathways that operate during early *T*. *cruzi* infection in human colonic epithelial cells. We investigated the phosphorylation activity of several axes of signaling cascades that may mediate *T*. *cruzi*-induced colon pathology. Our results showed that the parasite significantly altered the pattern of phosphorylated kinases and phosphoprotein levels during infection. Specifically, we found that *T*. *cruzi* deregulated the phosphorylated levels of 21 kinase phosphorylation sites, in the infected cells at different time points compared to uninfected controls. Bioinformatics analyses revealed that these phosphoproteins can be mapped to a variety of signaling pathways including those involved in neuronal, inflammatory, and fibrotic responses. Currently, it has been proposed that intense degenerative inflammatory changes occur in chagasic megacolon tissues, accompanied by increased collagen deposition and fibrosis. These changes are thought to mediate neuronal damage as well as intramuscular denervation, leading to chagasic megacolon [7]. The bioinformatics network developed in our study revealed significant alterations in the phosphoproteome associated with the signaling pathways that may cause the colon pathology observed in chagasic patients. The altered phosphoproteins we observed in our study have been reported to play a role in cellular pathways that can cause tissue fibrosis, neuronal damages, and negative immunological responses. The upregulated phosphoproteins involved in neurological and inflammatory pathways can damage colonic myenteric neurons during *T*. *cruzi* infection [20]. Bioinformatics analysis of array data also showed an overall increase in the proinflammatory response in HCoEpiC during *T*. *cruzi* infection. Hence, we further analyzed the associated proinflammatory pathways and found that NF-kB and JAK2-STAT1 pathways are activated by *T*. *cruzi* infection. The increased level of NF-kB (p-NF-kB, p-IKKα/β, p-TAK1 and p-IRAK4) revealed the high probability of activation of Toll like receptors by *T*. *cruzi* infection of HCoEpiC [21]. The upregulated level of JAK2-STAT1 pathway in HCoEpiC also correlates with the activation of interferon signaling pathway during *T cruzi* infection. Our observation complements previous studies suggesting the activation of NF-kB and JAK-STAT signaling pathways by *T*. *cruzi* [22–24].

Some of the phosphoproteins modulated in array during the infection, including p-c-Jun, have been correlated with increased expression of TSP-1. Previous reports show that the transcription factor c-Jun enhances TSP-1 promoter activity resulting in increase in TSP-1 [25, 26]. These findings support our previous reports showing that the parasite increased the level of TSP-1 during cellular infection [27]. Increased TSP-1 can mediate fibrotic responses [28] including those seen in megacolon [29]. The above observations support our findings that the level of TSP-1 increases during *T*. *cruzi* infection of colonic epithelial cells. This increase in TSP-1 protein is inhibited by a JNK inhibitor SP600125 suggesting that TSP-1 protein increase is regulated by p-c-Jun during *T*. *cruzi* infection (Fig 5C and 5D).

In the current study we focused on transcription factors CREB and c-Jun whose dysregulation have been suggested to be involved in several major pathologies. Deregulated levels of p-CREB have been reported in brain pathology [30] and defects in neuronal homeostasis [31]. c-Jun has been reported to play a role in sclerosis [32], neuronal defects [33], and several other pathologies.

Our array data showed significant upregulation of p-Akt S473 in *T*. *cruzi*-infected colonic cells compared to uninfected control. Our data also showed a significant increase in upstream p-JNK and downstream p-c-Jun during *T*. *cruzi* infection. This is important as an increase in JNK phosphorylation has been reported to play a role in endothelial dysfunction [34]. The activation of JNK followed by CREB as shown in our study was previously reported to be important in enhancing the transcription of sphingosine kinase 2 (SPHK2), which is elevated in colon cancer [35]. Studies have also shown that RSK1 may regulate CREB phosphorylation to perform various biological functions [36, 37]. Our data emphasized the potential role of CREB and JNK in causing the colon pathology observed in some *T*. *cruzi*-infected individuals. Our data also showed the upregulation of p-HSP27, an anti-apoptotic protein [38] and HSP60, a molecular chaperone which facilitates cyto-protection through stabilization of mitochondrial proteins [39] during *T*. *cruzi* infection. These proteins collinearly matched the increase in Akt phosphorylation in the infected cells. Our results show upregulation of p-HSP27 during *T*. *cruzi* infection, in agreement with other reports, suggesting that *T*. *cruzi* infection enhances cell survival [40, 41]. Additionally, Akt activation in stressed cells results in HSP27 phosphorylation [42], and the prosurvival effect of this protein occurs through its interaction with Akt.

Interestingly, c-Jun and CREB play a role in the expression of several proteins that are essential in regulating extracellular matrix (ECM) protein interactions [43, 44]. The deregulation of both these phosphorylated proteins during early *T*. *cruzi* infection results in immune modulations and fibrogenic responses. These fibrogenic responses increase fibrosis and muscular hypertrophy reported in the colonic sections from Chagas disease patients suffering from megacolon [8, 9, 45]. For instance, p-CREB has been reported to transactivate TGF-β1 expression to mediate hepatic fibrosis [46]; it also plays a key role in several immune responses [47]. JNK signaling plays an integral role in several key mechanisms involved in fibrogenic responses via direct phosphorylation of SMAD3, a profibrotic transcription factor [10, 48]. We also previously explained how ECM proteins are involved in *T*. *cruzi*-mediated Chagas pathology [11, 49]. CREB promotes an antiapoptotic survival signal in macrophages, leading to enhanced host immune responses during infection [50, 51]. Others also showed that CREB phosphorylation can induce TNF-α production during bacterial infections [52]. Activated CREB promotes a survival signal response in macrophages [47], leading to immune inflammatory responses during *T*. *cruzi* infection. Additionally, c-Jun has diverse functions in colon disease as well as in cancer progression [53, 54].

**Fig 9:**
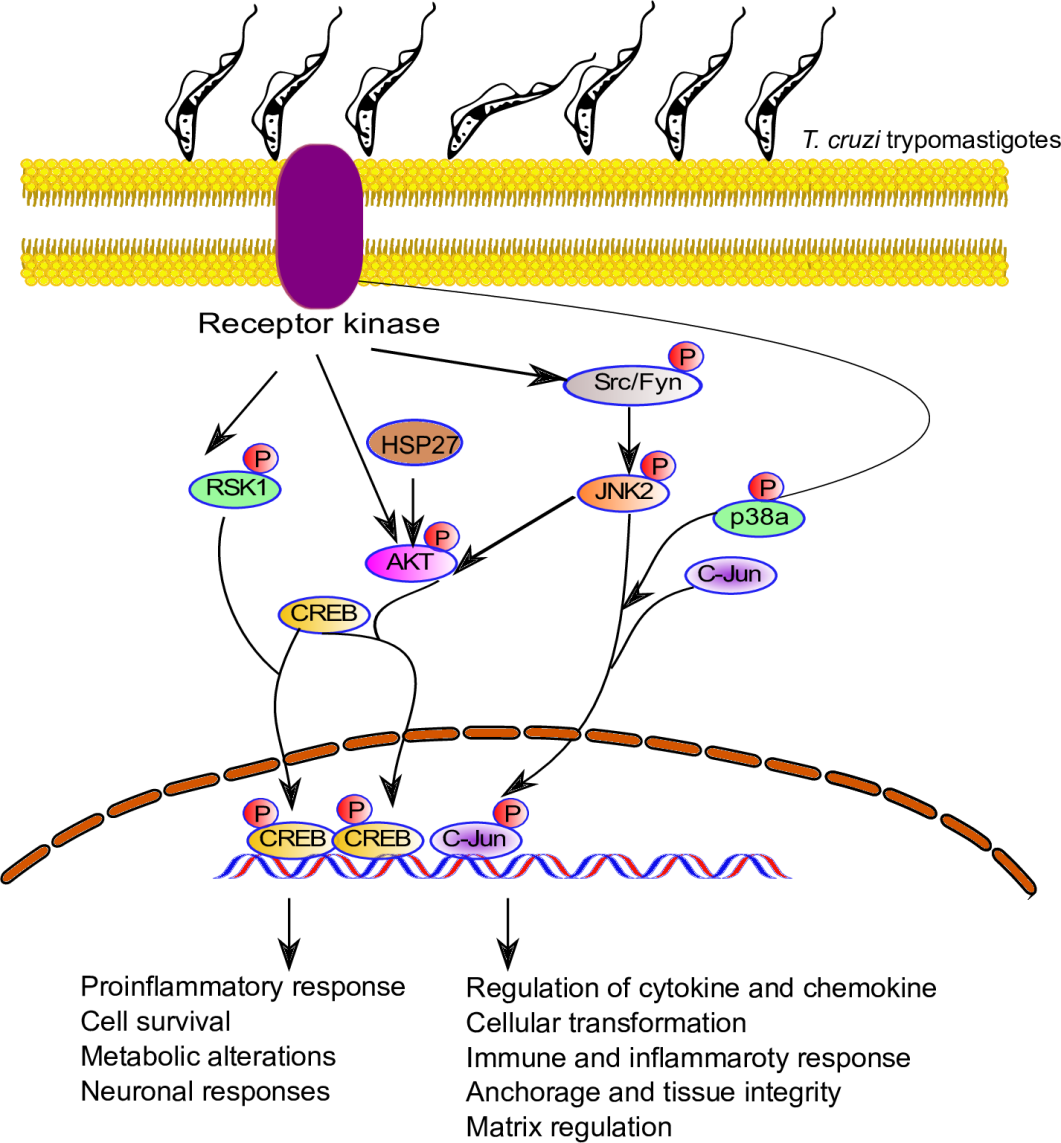
Proposed model of signal transduction pathways triggered by *T*. *cruzi* infection in primary colonic epithelial cells. *T*. *cruzi* infection of primary colonic epithelial cells led to the phosphorylation of several kinases and proteins, including Akt, RSK, and JNK. These kinases have been reported to phosphorylate c-Jun and CREB. The phosphorylated transcription factors are translocated into the nuclei of infected cells, as shown in Figs 7 and 8. The identity of receptor(s) involved in this process remains to be elucidated.

Data from our phosphoproteomic assays showed a significant increase in Src phosphorylation. These observations support a previously published conclusion that the remodeling of the ECM occurs through increased focal adhesion kinase and Src signaling [55]. Therefore, we postulate in our model that this may be one of the ways that *T*. *cruzi* causes pathology observed in chagasic individuals (Fig 9).

Our data show that *T*. *cruzi* infection of colonic cells significantly enhanced the phosphorylation of c-Jun and CREB in a time-dependent manner during the early phase of infection. Furthermore, nuclear colocalization of both these activated transcription factors increased in the infected cells compared to uninfected controls. Our data complement findings from previous studies showing that CREB binds to consensus cAMP-response element (CRE; TGACGTCA) and the overlapping activator protein-1 (AP-1) motif (TGACTGA, designated CRE2/AP-1) [56]. The phosphorylation and nuclear colocalization of both CREB and c-Jun during early *T*. *cruzi* infection may promote heterodimer formation between these two transcription factors and/or competitive binding during *T*. *cruzi* infection. Therefore, these transcription factors may play a novel role in mediating molecular deregulations in *T*. *cruzi*-infected colon cells. However, the detailed mechanisms remain to be deciphered.

Our model suggests that *T*. *cruzi* increases cellular levels of phosphorylated kinases, thus activating transcription factors including p-c-Jun and p-CREB that are all translocated into the nucleus to regulate inflammatory responses and cellular transformations. The colocalization of p-c-Jun and p-CREB in HCoEpiC challenged with *T*. *cruzi* is a novel observation in the context of *T*. *cruzi* infection (Fig 9). It has been previously reported that p-c-Jun and p-CREB cross-talk to cause cellular transformation [56–58]. Furthermore, increased TSP-1 expression induced by increased p-c-Jun can lead to a fibrogenic response which can cause colon pathology that could be implicated in the onset of megacolon. Fibrogenesis, due increased deposition of TSP-1, can lead to a marked thickening of the colon epithelium as observed in tissue sections of specimens from chagasic megacolon patients compared to controls [59]. Overall, we conclude that the colocalization of upregulated p-c-Jun and p-CREB is a putative key feature in *T*. *cruzi*-mediated colon pathology, warranting further investigation.

## Supporting information

S1 Fig Phosphoproteomic array analysis of HCoEpiC in starved conditions at 0, 60, 90, 120, 180 minutes. (DOCX)

S2 Fig Inhibition of p-JNK and p-c-Jun in HCoEpiC by SP600125. The results show the inhibition of p-JNK and p-c-Jun at all time points compared to control.
(DOCX)

S1 Table List of all 43 kinase phosphorylation sites and their relative fold change at different time points compared to uninfected control. (XLSX)

S2 Table List of all 43 kinase phosphorylation sites and their relative fold change at different time points in the absence of parasite compared to control. (XLSX)

S3 Table List of canonical pathways involved in *T*. *cruzi* infection as revealed by Ingenuity Pathway Analysis (IPA). (XLSX)
