# Supplementary material for: Phospho-proteomic analysis of primary human colon epithelial cells during the early Trypanosoma cruzi infection phase
Source: PLoS Negl Trop Dis. 2018 Sep 17;12(9):e0006792. doi: 10.1371/journal.pntd.0006792 (PMC6160231; doi:10.1371/journal.pntd.0006792)
Supplement: S1 Fig — (DOCX) [file pntd.0006792.s001.docx]

**Supporting Information**

**S1 Figure:** Phosphoproteomic array analysis of HCoEpiC in starved condition at 0, 60, 90, 120, 180 minutes.


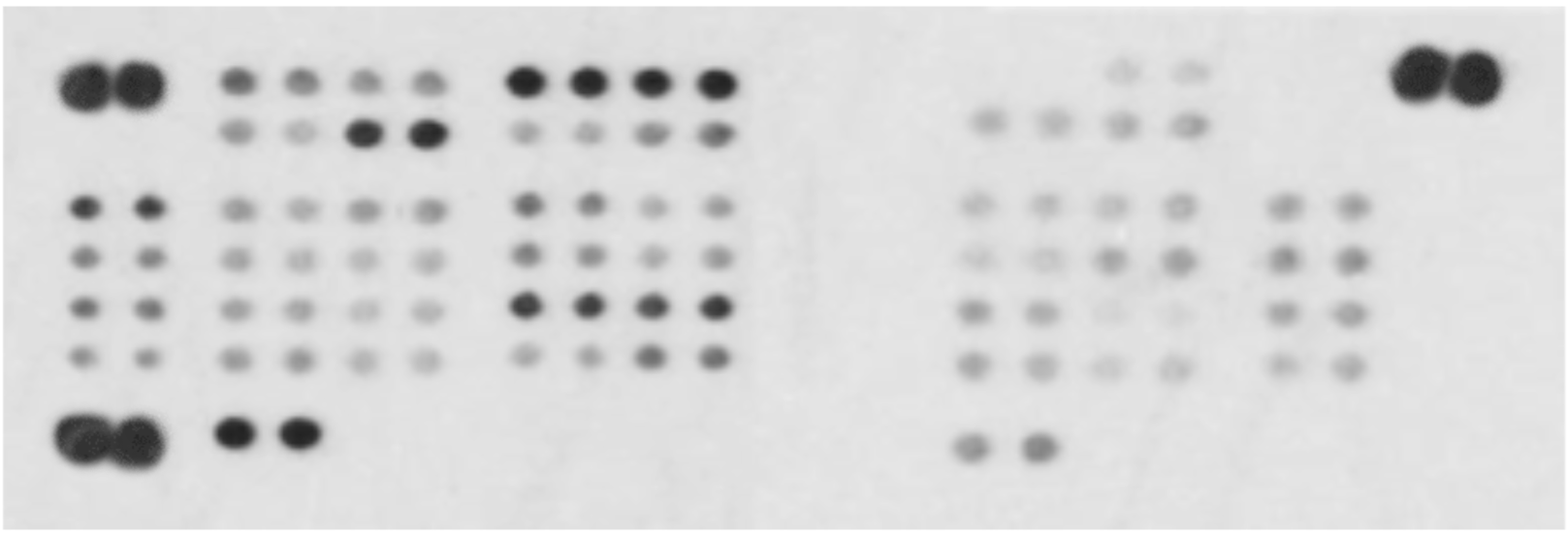

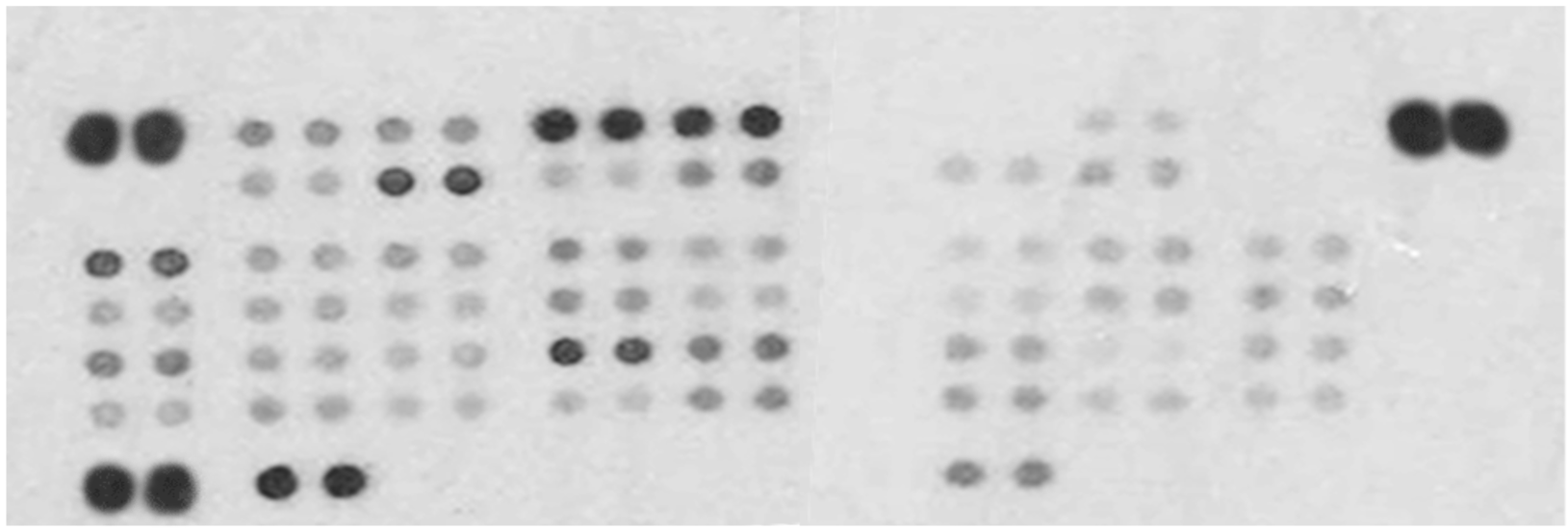

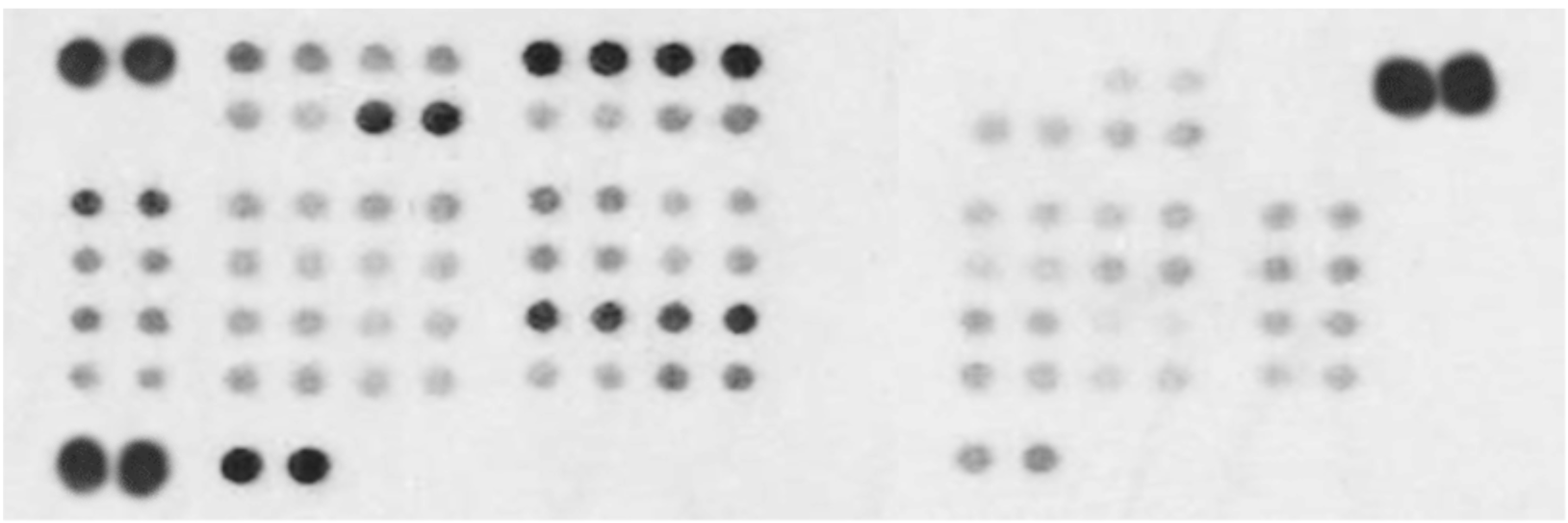

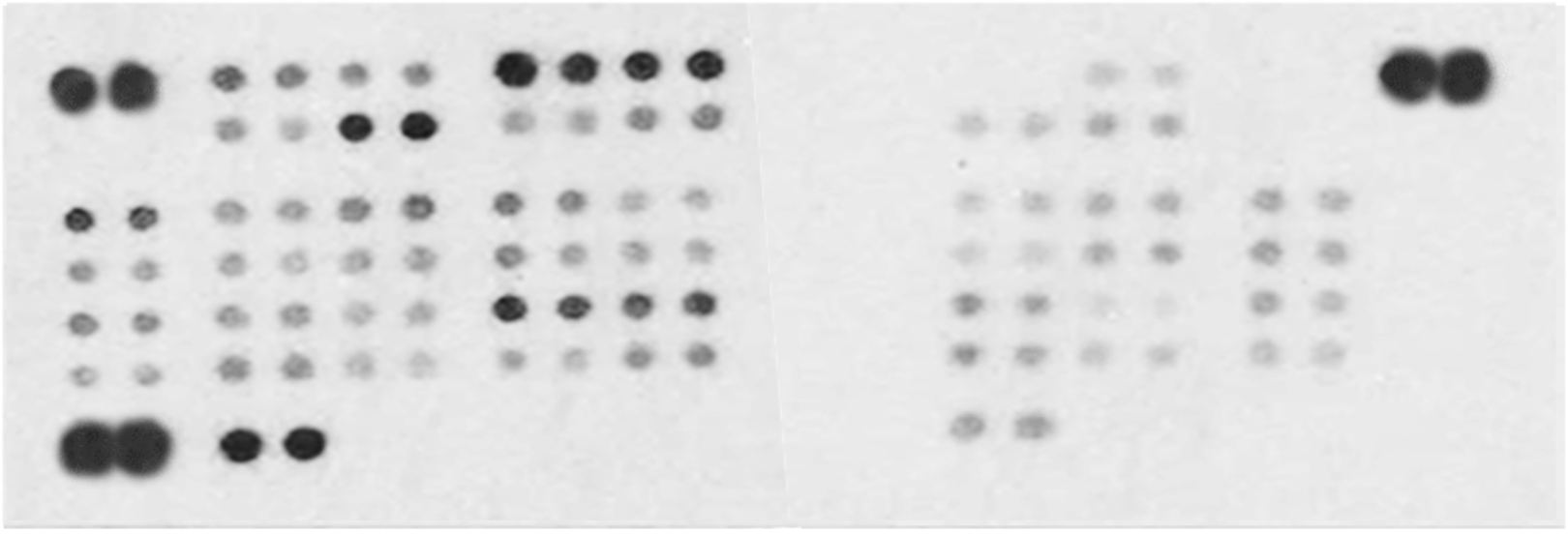

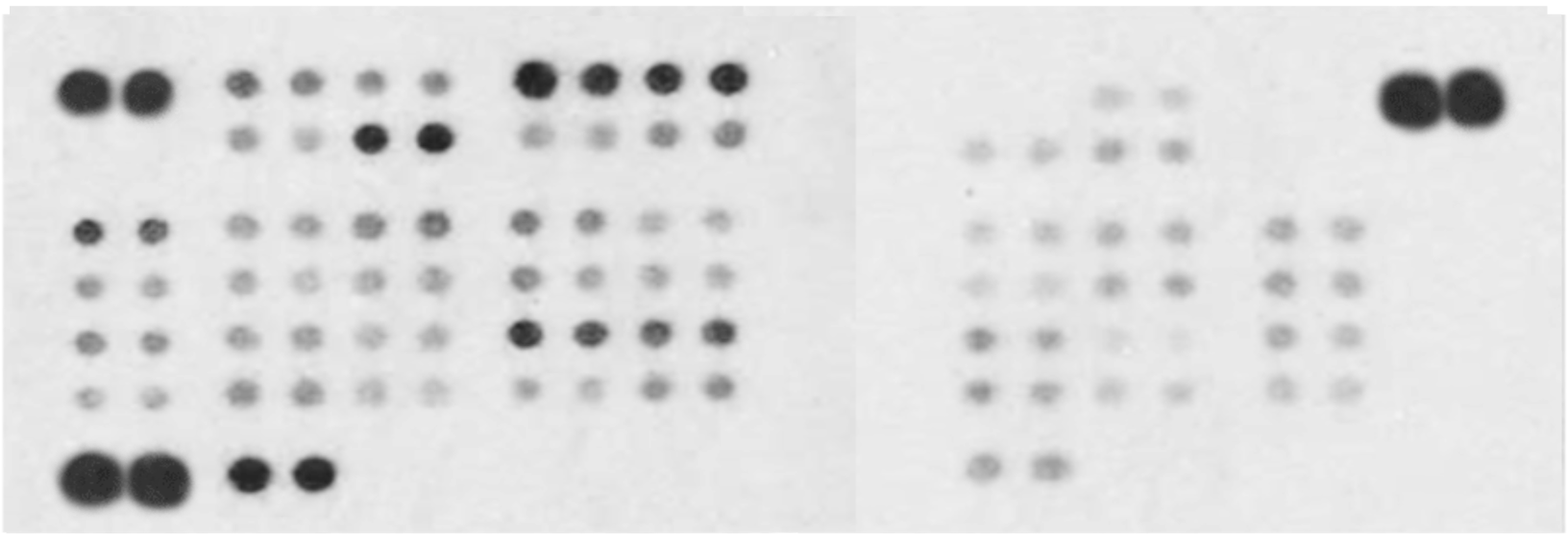


0 min

60 min

90 min

120 min

180 min
