# Supplementary material for: Phospho-proteomic analysis of primary human colon epithelial cells during the early Trypanosoma cruzi infection phase
Source: PLoS Negl Trop Dis. 2018 Sep 17;12(9):e0006792. doi: 10.1371/journal.pntd.0006792 (PMC6160231; doi:10.1371/journal.pntd.0006792)

**Supporting Information**

**S2 Figure:** Inhibition of p-JNK and p-c-Jun in HCoEpiC by SP600125. The results show the inhibition of p-JNK and p-c-Jun at all time points compared to control.


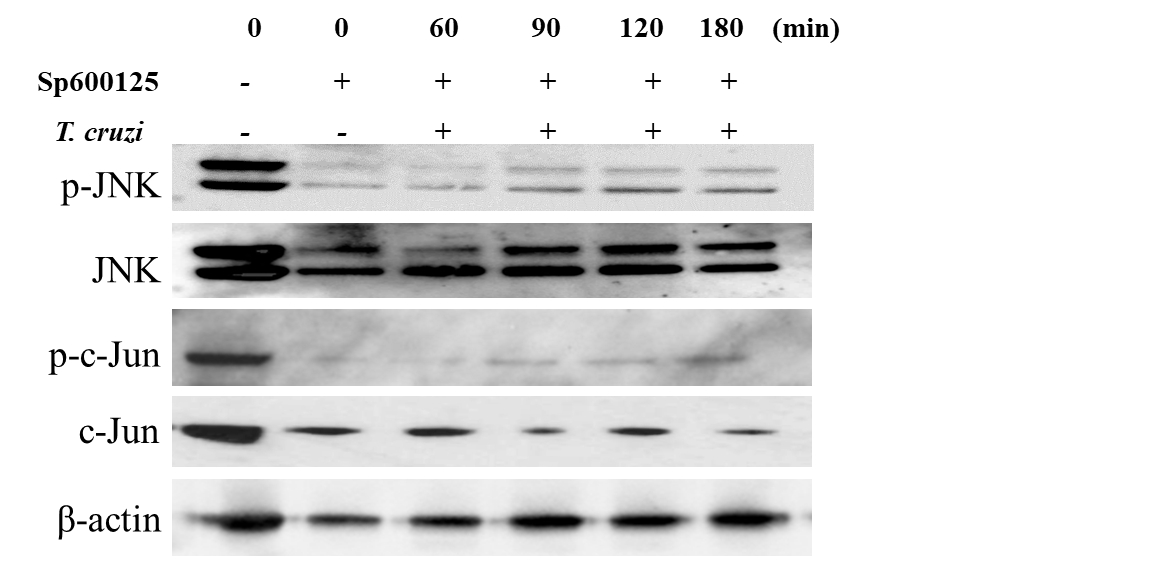

Supplement: S2 Fig — The results show the inhibition of p-JNK and p-c-Jun at all time points compared to control. (DOCX) [file pntd.0006792.s002.docx]
